# eHealth initiatives; the relationship between project work and institutional practice

**DOI:** 10.1186/s12913-019-4346-0

**Published:** 2019-07-24

**Authors:** Line Lundvoll Warth, Kari Dyb

**Affiliations:** 1 Norwegian Centre for E-health Research, P.O. Box 35, N-9038 Tromsø, Norway; 2 University of Tromsø, The Arctic University of Norway, Hansine Hansens veg 18, N-9019 Tromsø, Norway

**Keywords:** Project work, Institutional practice, Summary care record, E-prescription, Norway, Implementation, Project managers, Qualitative analysis

## Abstract

**Background:**

Large-scale, national eHealth services, such as the summary care record (SCR) and electronic prescriptions (e-prescriptions), have been implemented by project managers as Norwegian health authority initiatives. Few studies have been conducted on the large-scale implementation of eHealth services and the relationship between the implementers’ work and the use of the tools in healthcare practices. Hence, there was a need to determine the project work with a focus on changes in practice. This study explores the implementation of the SCR and e-prescriptions from the perspective of project managers; how does the implementation work by project managers relate to institutional practices in large-scale initiatives?

**Methods:**

Twenty-two semi-structured interviews were held with project managers in 2016 and 2018 and were recorded, transcribed, and coded according to the content. The analytical concepts of the “project” and “practice” were used to focus on tensions between the dimensions of time connecting historically established social practice and in situ actions.

**Results:**

The eHealth initiatives were demonstrated to have been implemented as a part of the national strategy and achieved through close collaboration with the Norwegian Directorate of eHealth (NDE). Tensions arose in relation to task-oriented actions during the implementation of the project and the daily management thereafter. Further, the work tasks of the project managers were related to the dissemination of the tools while, in practice, the tools were related to actual use by professionals. The implementation of several projects simultaneously created tensions between the implementation of a tool and a specific practice, as well as between tools.

**Conclusion:**

The objectives set out by the project managers in relation to their work should be viewed as temporary, whereas a long-term objective should apply to the use of the tools. Hence, the work of implementing eHealth initiatives might call for a renewed definition of the empirical object. Identifying factors that affect uptake, such as gaps between the intended use of an object and in situ actions or historically established activities, might expedite the future success of national eHealth initiatives. The social aspect of institutional practice has a direct bearing on the potential of a project to be implemented successfully.

## Background

The term “eHealth” refers to the intersection of medical informatics, health services, public health, business, and information delivered or enhanced through information and communication technology [1]. Many promising eHealth implementation initiatives, such as introducing technology in healthcare, are characterized by non-adoption or abandonment by individuals and/or organizations. The barriers to and facilitators of how new tools are implemented are important considerations, together with resistance from professionals, as it is assumed that their attitudes toward the tools are pivotal to their success [2]. Several initiatives fail because they are not integrated into the organization and workflow [3], which has unintended consequences [4, 5]. Factors concerning individuals and implementation processes must be evaluated when considering the application of an initiative [6]. The initiative must fit the existing organizational goals and staff skill sets as well as improve patient-professional interactions and relationships between professionals [7]. The right distribution of stakeholders who are involved in the dissemination of a national eHealth initiative is necessary to secure its successful implementation [8]. Although project managers are directly involved in the implementation of such projects, only a few studies have been conducted to obtain their perspectives [7, 9, 10]. The perspectives of project managers are invaluable because they can identify factors that contribute to the success or failure of a new system. An exploration of the relationship between the work performed by project managers and the use of the tools in healthcare practices might contribute to a greater understanding of what is required for its successful uptake within healthcare organizations.

In Norway, as in many other countries, national policies support large-scale services and standards. In 2016, the Norwegian Directorate of eHealth (NDE) was established as a subordinate institution of the Ministry of Health and Care Services. The NDE is responsible for the implementation of national policies concerning eHealth in Norway, establishing the requisite standards, and steering and coordinating eHealth in close collaboration with national, regional, and local health authorities,; technical organizations, and other stakeholdersv. The Strategy and Action Plan for eHealth 2017–2022 [11, 12] describes the goals of a digitalized, collaborative healthcare service with a view to being simpler, better, and more holistic for Norwegians. As part of the national strategy, the NDE strives to establish the requisite standards and administer the use of the eHealth methodology nationwide [13]. In addition, it focuses on cooperation among interested parties (e.g., project managers) for the successful implementation of digital solutions.

The NDE is responsible for the development and implementation of the two large digital eHealth services in Norway: the Norwegian summary care record (SCR) (in Norwegian, *Kjernejournal*) and electronic prescriptions (e-prescriptions) (in Norwegian, *e-resept*), [13] both of which support the national strategy for eHealth in Norway. SCR is a new electronic service providing and containing key patient information. E-prescriptions are computer-based, electronic filling-in and transmission of medical prescriptions, replacing paper and faxed prescriptions. Project managers were hired to ensure the successive implementation of these services. The users described e-prescriptions as a success, while the SCR had a lower uptake. This discrepancy awakened our interest in understanding attitudes toward the tools by exploring the implementation of the SCR and e-prescriptions from the perspective of project managers. Project managers are well placed to contribute new, important knowledge to this field owing to their experience and expertise gained through direct involvement in planning and managing the implementations as well as being collaboration partners with the NDE.

Project management is based on various approaches [14–17] that emphasize planning and control dimensions [17]. From a broad project perspective, [18] it is important to ensure the fitness of a project for its political context, i.e., in terms of organizational strategy, managership, and stakeholder management. These approaches are task-oriented and have the potential to change standard practice. Drawing on cultural-historical activity theory (CHAT) [19] as a framework (see p. 13), we will broaden these perspectives and assess the actions and engagement of the project managers against the overall historical activity in which they were a part [20]. Thus, the relationship between the work of project managers and the use of the tools in the institutional activity was assessed in the current study.

It is valuable to manage innovations in healthcare as small projects, because behaviors and attitudes among professionals are central to their outcomes. Andreassen et al. [21] noted that small innovative projects create enthusiasm, local engagement, and commitment, as well as facilitate the alignment of policy and practice. Therefore, from a managerial perspective, there are benefits to organizing an information and communication–technology (ICT) innovation in healthcare through a small innovation project [21]. Despite the advantages of small projects, however, national policies generally support large-scale eHealth initiatives, even though it has been demonstrated in the literature that it is particularly difficult to implement them [5, 22] and national digital tools [23, 24]. Hence, there is a need to assess factors beyond the individual behaviors and attitudes of enthusiastic healthcare professionals. In this regard, illuminating the work performed by project managers for large-scale initiatives related to changes in practice will better elucidate the relationship between policies, project work, and social practice.

Thus, the work of project managers for the two large-scale implementation projects in Norway (the SCR and e-prescriptions) was considered in relation to the use of digital tools within the institutions’ organizational activities. Our study has previously demonstrated that the infrequent use of the SCR was attributable to a lack of trust in the SCR content by end users, [25] thus exemplifying resistance by doctors to the implementation of a large-scale initiative. Despite the low uptake, the project managers described the project as a success [26]. These results indicate a gap between perceptions of success, i.e., between those of the project implementers and those of the users. To further enhance an understanding of the implementation of large-scale eHealth initiatives, an assessment was performed of the relationship between the project managers’ work and the use of the project tools within the organizational activity. In this paper, we ask the following: how does the implementation work by project managers relate to institutional practices of large-scale initiatives? The intention was not to explore the technical aspects of the system itself, but rather to understand the relationship between the project managers’ work and the actual use of the tools.

### The summary care record and e-prescriptions

Norway has 5.3 million inhabitants and is geographically divided into four healthcare regions: the Central Norway Regional Health Authority, the Northern Norway Regional Health Authority, the Southern and Eastern Norway Regional Health Authority, and the Western Norway Regional Health Authority. Healthcare itself is organized into specialist services and primary care. Primary-care doctors consist mainly of general practitioners (GPs), while specialist healthcare services include public and private hospitals, private specialists, mental healthcare, specialized drug treatment, and ambulance services. GPs and specialists in primary and specialist care have access to both the SCR and e-prescriptions. Medical prescriptions are handed to patients by requisitioners, i.e., GPs and specialists. Largely, e-prescriptions have replaced paper prescriptions—in 2018, approximately 90% of prescriptions were processed as e-prescriptions [27].

The SCR is a national digital tool that grants access to selected health information to healthcare professionals, regardless of where a patient is treated. GPs have to register this information in the SCR to improve patient safety, as it permits health-care professionals to gain rapid, secure access to core structured data on each patient. The SCR is the first national digital tool that facilitates the sharing of patient information across all institutions and levels of care in Norway [28, 29]. At the end of 2017, each Norwegian citizen had a personalized SCR. Prior to implementation of the SCR, the four healthcare regions, including their primary care and specialist services, were not linked by a common information system. Despite the substantial financial investment and resources that have been devoted to its development, implementation, and deployment, the SCR is still not routinely used in the Norwegian healthcare sector. It is estimated that only 4–5% (250,000) of inhabitants have information to be registered in the SCR. By the end of 2017, critical core information had been registered with the SCR for only 0.4% (21,000) of inhabitants [30].

E-prescriptions are a tool for the exchange of secure prescription information between those prescribing and distributing medicines, and it is applicable to different levels of healthcare. E-prescriptions replace paper and faxed prescriptions. They are sent to a central database from which pharmacies and surgical stores can obtain their patients’ prescriptions by inputting their national identity numbers. This central database supports the exchange of information and reduces the risk of errors in medicine prescription and distribution. It also provides an overview of all medicines, which is particularly beneficial to patients taking several medicines at once (e.g., older people with complex conditions). In 2017, e-prescriptions were implemented fully in Norway’s primary and specialist services. Patients can now collect their prescriptions from any pharmacy or surgical store in the country via the central database. Thus, in support of the SCR, e-prescriptions constitute the second key aspect of the Norwegian national eHealth initiative.

## Method

Empirical data were collected using a qualitative research method in a case study of the implementation of large-scale eHealth initiatives in Norway [31]. A case-study approach enabled us to conduct in-depth research and develop concepts to interpret a historically and culturally determined phenomenon, i.e., the project managers’ work and institutional practices. Two study units were selected: the SCR and e-prescription. The study was divided into two phases, as it received financial support in different periods; the SCR was the first phase and e-prescriptions were the second phase.

The aim was to illuminate the relationship between the project managers’ work and the use of the eHealth tools in institutional practices by developing descriptions of the project managers’ implementation of the SCR and e-prescriptions in specialist services. We received an overview of all the project managers working with the implementation of SCR from the NDE. We decided to interview a minimum of two project managers from each healthcare region and three project managers from the Southern and Eastern Norway healthcare region, as it contains the most healthcare trusts. The data consist of interviews with project managers working with implementation in specialist healthcare services in all four healthcare regions in Norway (see Table 1 below).

**Table 1:** Empirical data

Regional Health Authority	Central Norway	Northern Norway	Southern and Eastern Norway	Western Norway
Healthcare trusts	5	5	15	5
Operating hospitals	9	4	9	9
First phase (September 2016); Summary Care Record				
Project managers	4	4	5	9
Project managers interviewed	2	3	3	4
Second phase (April to October 2018); e-prescription				
Project managers interviewed	2	3	3	2

Based on the NSD information, we randomly selected two or three project managers from each region, contacted them by email with information about the study, and invited them to participate in the study. We received some answers immediately while others needed gentle reminders. As several project managers had moved on to other positions and projects after working with SCR or e-prescription, we randomly selected another if one did not respond in a couple of days. In cases where the project managers replied after we had recruited our planned number of informants, we included them in the study. For that reason, there were more informants than planned.

In the case of e-prescription, there was no list or overview from the NDE of all the project managers. We used knowledge from the interviews with the SCR project managers as a starting point to acquire information about the project managers who helped implement e-prescription. When recruiting informants for the second phase (e-prescription), we mapped this knowledge onto the four healthcare regions and strove for the same number of informants in each region as the first phase using a “snowball” technique [32].

In total, we conducted 22 interviews with project managers. In the first phase of the study, accomplished in September, 2016, we interviewed 12 project managers who were responsible for the implementation of the SCR. Between April and October 2018, we interviewed 10 project managers who were accountable for the implementation of e-prescriptions. The first author conducted the 12 interviews with the project managers involved with the implementation of the SCR; as the study scaled up in its second phase, a research assistant was hired to conduct the 10 interviews with the project managers involved with the implementation of e-prescriptions. In total, 20 interviews were conducted by telephone, Lync, or Skype, depending on which technology the project managers used in their workplace. Two face-to-face interviews were conducted because the interviewees and the interviewer were based in the same city.

The interviews were semi-structured with predefined themes and subjects [33]. The informants were initially asked to (1) explain their background and the reason for being a project manager, (2) describe their work tasks as project managers and the ways in which the SCR and e-prescriptions were implemented (i.e., the project organization), (3) describe their collaboration with other project managers and implementation projects, (4) describe the challenges they encountered, (5) evaluate the goal achievement and the lessons learned in relation to the implementation of a similar initiative in the future, and (6) consider whether there were other relevant subjects which had not been discussed. Themes that they introduced in the interviews were followed up with questions when appropriate. Both interviewers were experienced in the field, offering the opportunity to capture rich, descriptive data. Each in-depth interview lasted approximately 45–80 min. The interviews were recorded, transcribed verbatim, thoroughly read, and coded according to the themes and content. The findings were then discussed by three senior researchers. The results in this paper reflect the patterns that emerged from the findings [34, 35] in relation to the implementation of the SCR and e-prescriptions.

We applied for approval from the Regional Committee for Medical and Health Research Ethics, but it was not required for this study. The data-protection officer at the University Hospital of Northern Norway did approve the study, and all the project managers who participated signed an informed consent form, which was sent and returned by email.

The results are analytical generalizations, [32] i.e., a combination of a theoretical point of departure (CHAT), the empirical analysis itself, and the discussion of findings related to other studies. Hence, the findings are generalizations in a theoretical and empirical debate. [32] Generalizations are based on in-depth research in a context where the conclusions can provide an explanation of a widespread phenomenon. Through the selection of cases—two large-scale initiatives in Norway—we were able to study units where implementation occurred in the same historical period. The patterns of the project managers’ work are presented in the language of a cultural-historical framework (CHAT) [19]; hence, the results offer insight into the characteristics of the relationship between the “project” and the “practice” (see p. 14) of the implementation of large scale eHealth initiatives in Norway in this historical period.

### Reliability

In September 2016, the NDE invited all the project managers to attend a common national meeting on the SCR. We observed the meeting, in which 18 project managers participated. The aim of the meeting was to facilitate an exchange of experiences and a discussion among the project managers on how to increase the use of the SCR in specialist services. The interviews with the SCR project managers were carried out around this time, so our observation could verify the reliability of our interviews. We made notes and, afterward, compared them with the content of the interviews. In qualitative studies, reliability is an indication of the precision [36] and accuracy of the findings [33] and to what extent they could be reproduced if they were collected at another point in time by other researchers. The actions and claims of the project managers at the meeting appeared to be consistent with those in our interviews. Some of the participants at this meeting, in fact, had been interviewed several weeks earlier. Thus, the findings were deemed reliable.

This paper is part of a larger study that includes interviews with 25 GPs with access to the SCR (and e-prescriptions), nine interviews with NDE representatives who were involved in the implementation of the SCR and e-prescriptions, and a final document analysis. Some of the results have been presented previously [25, 26] and were discussed with stakeholders in the field. The interviews with the NDE representatives were conducted after those with the project managers on the SCR and e-prescriptions. Thus, the reliability of the themes that emerged from both the interviews on the SCR and e-prescriptions with the project managers and those with the national authority’s representatives could be confirmed.

All of the interviews were transcribed verbatim. Certain transcription excerpts are presented in this paper so that readers can assess the results and form their own opinions about the reliability of the information, despite a lack of access to the raw (sound) material. The excerpts are presented with references to the SCR (first phase) and e-prescriptions (second phase). Some interviewees were responsible for the implementation of both tools, but they are cited in this paper in accordance with the study for which they were recruited as informants.

### Framework for analysis of the implementation work

The analysis was inspired by CHAT [19] and an understanding that social activity is mediated by cultural tools. The activity was considered from the perspective of a dialectical relationship between the direction of the activity and how the object of the activity occurred, i.e., the objectives behind the project implementation and everyday institutional practices, respectively. Using CHAT as a framework, the “object of activity” refers to the actions directed toward a goal, i.e., outcomes. Changes in social practice demand a shared understanding of the outcome and a shared object of activity. CHAT places emphasis on the systemic structure of an activity that produces events and actions that evolve over time [19]. An activity is reflective of a series of actions that professionals from a social practice perform in a particular situation. The connection between established social practice and in situ actions describes an interdependence between the historical and the “here and now,” respectively [37]. Generally, CHAT is used as a tool to analyze tensions between different actions in multiple activity systems that have the potential to change situated practices [19].

An important aspect of the analysis of the implementation of eHealth initiatives was to focus on the dimension of time with a view to understand the short-term actions and long-term activities as part of a timeline. We facilitate this by using the analytical concept of “project” to relate actions to project mangers’ work with implementation and of “practice” to refer to the use of the national digital tools in healthcare institutions. A “project” is a short-term action that intervenes in the established historical pattern of institutional activity. The work of the project managers was limited to actions that had to be performed within a relatively short time, and their proposals had to be established as part of the daily routine at the healthcare institutions, changing established practices and creating new ones. While a project concentrates on actions that unfold within a limited time span, a “practice” framework focuses on long-term, ongoing institutional activities. A project is task-oriented and often has the potential for change as an outcome of an activity, [38, 39] whereas practice is based on patterns of interaction and involves social relationships and collective learning over time.

## Results

The key themes identified from the interviews with the project managers who implemented the SCR and e-prescriptions are explained in this section. In attempting to determine how the project managers’ work implementing the tools at the healthcare institutions influenced the establishment of these large-scale initiatives, it was initially necessary to explore what the project managers did within the context of a project. The projects were demonstrated to have been implemented according to the national strategy and achieved through close collaboration with the NDE. The project managers were shown to be task- and time-oriented in their work, as they had to execute pre-defined work tasks to meet the overall purpose and goals inherent in a short-term project. As professional project managers, they often engaged in the implementation of multiple tools and projects simultaneously. As point of departure, the informants were recruited for the study for their positions as project managers. Out of 22 project managers, 13 were men and 9 were women, and 21 described themselves as experienced project managers.

### The collaboration between project managers and the Norwegian directorate of eHealth

The implementation of both the SCR and e-prescriptions was initiated by the Norwegian Ministry of Health. It was led by the project managers in each healthcare region and implemented at a local or central hospital for a predefined period. The project managers worked in close collaboration with the NDE, which is in the capital of Norway. The collaborative work consisted of regularly held meetings, supported work, and standard information for all healthcare regions. Some informants had the following to say about their collaboration with the NDE:

“I appreciate the national initiative and the collaboration with the Ministry of Health. A national perspective—I think that’s the direction to go.” Informant e-prescriptions 4

“The collaboration with the NDE has been quite close and intense. We had people from there supporting us, both for the SCR and e-prescriptions, with regularly held meetings with the NDE and the Ministry of Health.” Informant e-prescriptions 2

“We regularly collaborated with the NDE in the rundown.” Informant SCR 5

In addition to holding meetings and conducting follow-ups on the work of the project managers, the NDE produced standard information for distribution to patients and professionals, as one informant verified:

“The information was nationally produced. We inherited procedures from others that had to be adjusted to our health region. We had a lot of information brochures, in addition to the meetings [with professionals].” Informant e-prescriptions 10

In keeping with the national strategy objectives, the work of the project managers was guided by certain standards to ensure the standardized implementation of the eHealth methodology nationwide. The large-scale nature of the initiative benefitted the project managers, as their work was supported through collaboration and close liaison with the NDE and the originator of the project, the Ministry of Health. Cooperation was key to ensuring the successful implementation of digital solutions in agreement with the national directive.

### The task- and time-oriented professional project managers

The project managers were recruited for their competency in project management, and most of them were previously involved in the implementation of healthcare initiatives. Of the 22 interviewees, 21 described themselves as experienced project managers. Some of them worked full-time to implement the initiative, while others did so on a part-time basis. Some of them were recruited from private companies by large hospitals with the principal responsibility of implementing electronic information systems, as some informants verified:

“I am a hired project manager working for a private company. Both e-prescriptions and the SCR were implemented at the same time.” Informant SCR 7

“Basically, I was a project manager for e-prescriptions and then for the SCR since they had common features. I am working for XX [a private company] working on digital renewing.” Informant SCR 4

As these quotations illustrate, the project managers were experienced in the implementation of electronic tools in specialist services. In some hospitals, the same individual was responsible for the implementation of several projects (i.e., e-prescriptions and the SCR) simultaneously or within a short timeframe. He or she held the position for a restricted and predefined period and thereafter applied his or her expertise to other projects. On project completion, the technical department at each hospital took over the responsibility of maintaining the technological systems, as one informant verified:

“( … ) when the project period was over, professional competence regarding this project was passed on for other projects. ( … ) When you have a project, you get the focus, the opportunities, as a project manager.” Informant e-prescriptions 2

As this informant described, the eHealth initiative required the project managers to perform defined work tasks within a stipulated and limited timeframe. This illustrates how a project was organized into time-limited tasks. Specific competencies are thus required of project managers, and precise resources are allocated to the projects. Tensions arose in relation to task-oriented actions during the implementation of the project and the time thereafter. Once the project period is over, the daily management of the established institutional practice continued. Hence, we define the object of the project managers’ work as “temporary” during the project, and the technical department that relates the initiative to work tasks in long-term daily management.

### The establishment of pre-defined work tasks to realize the project goals

The project managers were responsible for preparing, providing information on, and implementing the necessary infrastructure to facilitate access by healthcare professionals to the SCR and e-prescriptions. They also encouraged practitioners to complete the e-learning courses for both the SCR and e-prescriptions and to pass the acceptance test needed to use the SCR, as this informant verifies:

“The number of SCR users indicates that we did not succeed ( … ). We hoped that all who took the test would use it [the SCR]. We have not succeeded in terms of the number of users ( … ).” Informant SCR 6

The numbers of practitioners who took the SCR test and those who logged on (i.e., potentially used the SCR) did not match. The e-learning test was mentioned as an indicator of the success of the introduction of the SCR, as well as of whether the training was successful, as one informant explains:

“We had a goal for how many took the test, not for how many logged onto the SCR.” Informant SCR 10.

Although the number of practitioners who took the e-learning course and passed the test did not correspond with the actual number of SCR users, all the SCR project managers were satisfied with their work during the project period. The project managers were asked to describe the goal of and success criteria for their work, that is, the implementation of the SCR and e-prescriptions. All of them stated that their main goal was to make the tools accessible to practitioners, as this informant indicates:

“It is a success because we have turned it on ( … ). We have prepared it so that those who want to use it can do so. But it is up to the clinics themselves to start using it.” Informant SCR 3

Others cited a more ambitious goal for the work:

“( … ) the goal is to start to use it. It does not say anything about volume or how often healthcare professionals should use it.” Informant SCR 11

The work tasks were designed to enable practitioners to pass the acceptance test, turn on the SCR, and help healthcare professionals to start using it. The extent to which it was used was a secondary consideration. Nevertheless, the project managers had access to the traffic data of the professionals who logged onto the SCR and could evaluate its success according to the extent to which it was used. A central identified tension was that the work tasks of the project managers were related to the dissemination of the SCR while, in practice, the tool was related to actual use by professionals.

The work of implementing new tools was also evaluated according to other criteria such as time and budget, as this informant indicates:

“There has not been any delay in time. It has not exceeded the budget; the SCR has been delivered before the deadline.” Informant e-prescriptions 1

As the above informant emphasized, the success of the project managers was specifically defined by their ability to deliver the project and to implement the tools within the allocated budget and set deadline. Hence, while it is traditional for a project manager to apply himself or herself to project-related tasks for a short-term period, the tool itself has a much longer life span. This indicates that the project defines one object of the work, while the healthcare professionals have other objectives, e.g., the treatment of patients.

When the project period for implementing the SCR and e-prescriptions was over, the rate at which the tools were adopted and used varied. The SCR was not utilized at the intended scale, but e-prescriptions were described as a successful tool on completion of the implementation:

“E-prescription is to put electricity on paper. It was requested and received almost red-carpet treatment. Welcome, finally! Handling the prescription electronically is easier than using paper. It has been an implicit success. SCR—that was harder. People did not have a relationship with it.” Informant e-prescriptions 1

“Huge interest in e-prescriptions and poor interest in the SCR. ( … ) This [e-prescription] is defined as a conditional success in our management, this implementation project.” Informant e-prescriptions 10

As can be seen in these quotations, the project managers considered the e-prescription project a success, as it was associated with considerable user interest. While various explanations were given for the reticent adoption of the SCR, the e-prescription tool was considered to have expanded the paper version for both professionals and patients. Both the implementation and use of e-prescriptions were described as successful. The project managers expressed satisfaction in this regard:

“( … ) as a tool for prescription, it’s [e-prescription] an absolute success. For me, as a project manager, it has been easier to implement ( … ) [than the SCR].” Informant e-prescriptions 5

This informant, who was project manager for both the SCR and e-prescriptions, found it easier to implement the e-prescription project because of the positive perceptions of the tool and its use among professionals. As an outcome, e-prescriptions constitute a historical change in practices, and they have established new work patterns between those who prescribe and distribute medicines. These results illustrate that users’ perceptions of the new tools significantly influenced the ease with which the project implementation could be executed. If an implementation project is well received, it makes the work of the project manager easier, as the users are more engaged. Conversely, the opposite is true. Those that are not well received by the users, on the other hand, make the project managers work harder to implement them.

### The simultaneous implementation of several projects at the same time

Mention was made during the interviews about the relationship between the eHealth initiative and existing electronic information systems in the specialist services. Plans for new electronic systems or recently implemented ones were shown to influence the work of the project managers. In some hospitals, the SCR and e-prescriptions were implemented simultaneously, often by the same project manager. There were limitations to the simultaneous implementation of the two systems:

“Simultaneous implementation is challenging because one of them had been waited on for years ( … ), while the other one is unfamiliar [to the practitioners]. They do not know how to use it in daily practice. ( … ). One [is] drowning in the other one.” Informant SCR 9

Simultaneous implementation was not only performed by the project managers; it was also a feature of their partners’ work (i.e., the computer retailers and the NDE). The latter developed several technologies and services, and this had to be managed by several project managers, as this informant indicates:

“The problem with national solutions today is parallelism. Both the system delivery and the Directorate [NDE] ( … ) work on the same projects, but not in the same direction.” Informant e-prescriptions 10

Thus, the introduction of new tools was seen to comprise separate projects that were characterized by different goals and tasks. The simultaneous implementation of systems meant they had to compete with one another, as this informant indicated:

“I would definitely not implement the SCR and e-prescriptions at the same time if I had a second chance. ( … ) E-prescriptions ran over the SCR ( … ).” Informant e-prescriptions 10

The implementation of multiple tools within a similar time period complicated the work of the project managers, as their introduction and use led to competition between them despit.

e their varying different purposes. A number of actions unfold within a limited time span when several projects are implemented simultaneously. Thus, the challenges were not only confined to the work of the project managers but also extended to tensions between the implementation of one tool and a situated practice, as well as tensions between the tools. Hence, the established historical pattern of institutional practice was impacted twofold.

## Discussion

CHAT [19] inspired how we assessed the relationship between the work of the project managers and the implementation of their tools (the SCR and e-prescriptions) in healthcare institutional practices, which affected the implementation of these large-scale initiatives. This assessment required an understanding of the tasks and functions of the project managers and thereafter to determine how their work was reflected in institutional practice. The dimension of time was used to identify how the object of activity occurred in the project implementation and the everyday institutional activities.

The project managers were hired as professionals in their field and recruited for their competence in project management. Some of their careers were so established that they had already implemented several other technology-based initiatives in healthcare in addition to the SCR and e-prescriptions. They described how they implemented the two tools using the same work methods, following an approach whose goal was to fit the project to the political context and to agree with the national strategy. They clearly had adequate knowledge of project management, supported by the expertise they gained from engagement in projects on an everyday basis, which ensured their competence in heading projects within a defined and relatively short period. The organizational and political importance of this knowledge was apparent. A key directive of the project managers was also to take their work from one point to the next while adhering to a predefined project plan. The dimension of time produced diversity, which affected how the project work unfolded and how the project managers related to the SCR and e-prescriptions as a work practice. When the project period was over, they were recruited to manage other projects. The implementation of the new eHealth tools could be perceived as a negotiation between the work practice of the users and the time limit of the project.

From the perspective of a short-term project, the project managers worked effectively to realize their overall purpose and goals. Their work can be described according to mainstream management theory, which refers to taking a predictable path according to a predefined plan [18]. The project and its status were judged according to whether the originally specified goals were achieved, and its success was determined according to whether certain project criteria were met (e.g., accessibility of the tools to users and the completion of e-learning courses and certification tests). The project managers’ engagement in implementing multiple tools simultaneously affected their work. Several projects competed for their attention, and the lack of adoption of the tools complicated their work tasks. Nevertheless, they ultimately described the project outcomes as successful, as their actions were driven by the plan and the goals were defined according to set work-task criteria (e.g., the delivery of e-learning courses and the administration of acceptance tests).

The extent to which the outcomes were deemed to have meet the original objective will be influential in determining which factors constitute success. The implementation of a new tool might meet the organizational criteria for success even if it is not normalized in practice [40]. The success of a project is not an objective measure. The objective of the entire process is alignment; in other words, any improvement increases the likelihood that a project will be considered successful [41] .The project managers might define their work as a success, whereas the use of the proposed tools in institutional practice might be more successful or less successful, as was the case the with e-prescriptions and the SCR, respectively. User perceptions of e-prescriptions were positive, and the tool was welcomed. This made the work of the project managers easier, as the users were more engaged. The managers and/or the individual engagement of the project managers, then, were not the sole reasons for the success of e-prescriptions.

By framing the findings in the context of “practice”, attention was drawn to work that involved the project considerations which, in this case, dealt with tensions between these goals and actual user practice. The tasks had the potential for change in practice. In the case of the SCR, fulfilling the project goal was insufficient to change professional practice to the desired degree. Adoption of the SCR tool required a change in established practices and the creation of new ones to facilitate information-sharing across different levels of care. The e-prescription tool was new but was used to perform the same task, that is, capturing the same actions within the same social practice and in the same situation. It involved the replacement of paper with an electronic tool in situ without changing the way in which the professionals collaborated. The healthcare practice of providing a prescription encompasses a series of actions that professionals perform in particular situations, like when a patient needs medicine. The established practice of prescribing medication using paper was simply replaced by an electronic action. The e-prescriptions are sent to a central database, which supports the exchange of information without depending on other professionals to make changes to their collective practices. Conversely, when using the SCR, the GPs have to register the information in the SCR, thereby establishing a new practice (i.e., information-sharing between different levels of care). A GP who is treating a patient is required to look up the relevant information in the SCR and thereby establish a new practice. Hence, the social aspect of institutional practice is a critical point for the successful project work and uptake of tools within organizational arrangements.

The introduction of eHealth technologies is associated with tension, owing to the existence of parallel workflows, and it is tied to the analytical concept of practice because it propels change in social practice. The rejection of a technology is linked not only to its achievements during project implementation, but also its goals and achievements thereafter. Thus, the tools must be adapted to the local work practice to accommodate new forms of social practice. A gap exists between, on one hand, the definitions of success by project managers and end users, and, on the other, between the historically established practice and the establishment of a new work practice. Although the project managers defined the project as a success in the sense that it met the project criteria, the use of the tools by users was infrequent. Hence, the differences in success between the SCR and e-prescription initiatives cannot be attributed to the work of the project managers but rather to collective learning over time [19, 37].

For smaller projects, local enthusiasm, engagement, and commitment, as well as the alignment of policy and practice, help to maintain the local activity [21]. The engagement of others who are a part of the institutional practice, who are part of the daily community, and who share the same object could also equate to a definition of success. Even if the implementation fails, local enthusiasts can advance the new ideas.

Although such systems are likely to be better accepted by local users than a standardized solution, endeavors that are determined by individuals or small groups of enthusiasts may not have longevity [22, 42]. As demonstrated in the present study, the implementation of a large-scale project was driven by professional project managers within a predefined period. The social relationship was not connected to the activity itself but rather to task-oriented actions. When the project period was complete, it became the responsibility of the healthcare professionals to perform the new activities using the SCR or e-prescriptions, especially as the project managers often had to leave the actual environment. Local enthusiasts, who often characterize local projects, might not be present.

Failure to implement technology in healthcare can be related to the characteristics of the technology itself; sometimes changes have to be made to the technology to satisfy user perceptions [22, 42]. Large-scale projects, such as national initiatives, are often subject to pressure to secure alignment, as in the present case, with the NDE simultaneously developing and implementing new functionalities for the SCR and e-prescriptions. The gap between project aims and the need for changes in actual social practices seems to continue with the emergence of new projects and the need to operate multiple tools within the healthcare field. Introducing new technological undertakings as projects seems to be a strategy that improves the probability of the project being perceived as a successful institutional activity in practice.

The study’s findings illustrate that the project managers adhered to the national strategy guidelines through close collaboration with the NDE. The interviews did not reveal any tensions in the follow-up with the NDE. However, as the objectives of the project managers regarding the work of the implementation of the eHealth initiatives might have varied among healthcare regions, the tension might have created an opportunity with regard to changes in institutional practice when multiple actions were directed toward achieving the same goals across different healthcare regions. The acquisition of an understanding of shared objectives and the impact of long-term activities (including institutional aspects that are historically established) is recommended. The implementation of complex tools to establish a new social practice is challenging. Conversely, there is a greater chance of success if the elements of social relationships and collective learning are present. Tools that create new social practice are more demanding, while in situ actions which do not involve social relationships and collective learning tend to be the most successful.

## Limitations

The implementation of two national eHealth initiatives in Norway, the SCR and e-prescriptions, was evaluated by interviewing the project managers in the current study. It has previously been demonstrated that long-term social practices are important when exploring institutional changes. The number of users of the SCR and e-prescriptions is indicative of the extent to which the tools are being used. Thus, a research design that permitted data collection in relation to the everyday uptake of the tools used for activities at the healthcare institution would have strengthened the study.

We conducted a study to obtain doctors’ perceptions of the SCR in 2016 [25]. Time is a key component of the ability to frame the practice of eHealth initiatives. Thus, a limitation of the current study was that the tools were not observed in practice over time, as this would have helped with an understanding of how meanings were created and how the interaction was organized and coordinated across different institutional levels. The observation of activities over time would have widened the knowledge base. Further studies are warranted in this regard.

## Conclusion

Policymakers in Norway have requested the large-scale implementation of eHealth initiatives in daily health service delivery [11–13]. Currently, the SCR and e-prescriptions are the national tools for communication- and information-sharing in Norway. Both have been implemented as NDE health initiatives with the objective of establishing the requisite standards, standardization, and administration of the eHealth methodology nationwide. The SCR and e-prescriptions are used in practice today, even though use of the former has been considerably less than expected by the health authorities prior to its implementation.

The failure of newly implemented technology can be related to the characteristics of the technology itself, as mentioned previously. When this is the case, changes are made to the technology to try to meet user perceptions, especially if there are adequate resources to effect such changes. Gaining an understanding of the experiences and perspectives of the project managers with regard to the implementation of the new system and their perceptions of which factors contributed to its success or failure are therefore useful albeit understudied. By exploring how the implementation by project managers relates to institutional practices in large-scale initiatives, we wanted to obtain an alternative understanding of the relationships between situated events (i.e., the project managers’ work and the eHealth initiatives in practice) to gain insight into the institutional changes involved.

Our findings illustrate how the project managers are doing their work according to their pre-defined plans. Hence, there are no individual explanations for failures in the work of implementing eHealth initiatives. While the project managers considered the project a success, use of the SCR was infrequent and less than anticipated. The SCR tool demanded changes to established practices and the creation of new ones to expedite information-sharing across different levels of care. The e-prescription tool, on the other hand, was developed to perform the same action in the same social practice, simply replacing the use of paper with an electronic tool and without changing the way in which healthcare professionals collaborate. In other words, the introduction of the SCR meant a change in social practice and the establishment of a new one while the introduction of e-prescription did not. Hence, the social aspect of institutional practice has a direct bearing on the ability of implementation to be successful.

The project managers’ objective was to fulfil the short-term project goals, in contrast to the objective of healthcare practitioners, whose objective was and is to deliver medicines (using e-prescriptions) and share information (using SCR), all of which depends on changes in collective practice over time. Hence, the work of implementing an eHealth initiative might call for a renewed definition of the empirical object of the implementation. The work of implementation is constructed from the individual project manager all the way to the social practice at the institutional level. This result might be an important input when establishing requisite standards and administering the eHealth methodology. The current study findings could be used as a foundation upon which the actions of project managers are based, but it is also necessary to go beyond these methods when attempting to understand the success or failure of the implementation of a tool in healthcare practice and to address implementation challenges better. The identification of factors that impact uptake, such as gaps among the definition of an object, the in-situ actions, and historically established activities, is recommended.

## Data Availability

The data that support the findings are held by Stein Olav Skrøvseth, Director, Norwegian Centre for E-Health Research, but restrictions apply to their availability. The data were used under license for the current study; thus, they are not publicly available. However, data can be obtained from the authors upon reasonable request and with the permission of the Norwegian Centre for E-Health Research.

## Abbreviations

E-prescriptions: electronic prescriptions
GPs: general practitioners
ICT: information and communication technology
NDE: Norwegian Directorate of eHealth
SCR: summary care record
